# Regulation of Autophagy in Aging and Disease

**DOI:** 10.1093/geroni/igaa057.2673

**Published:** 2020-12-16

**Authors:** Malene Hansen

**Affiliations:** Sanford Burnham Prebys Medical Discovery Institute, La Jolla, California, United States

## Abstract

The cytosolic recycling process of autophagy plays an important role in many age-related diseases and has been directly linked to aging, including in the nematode C. elegans where autophagy appears beneficially induced in many conserved longevity models. As a critical process to ensure cellular homeostasis, autophagy is regulated at multiple levels, yet it remains a challenge in the field to understand how the regulation of autophagy is integrated at the cellular and molecular level to ensure health- and lifespan benefits. I will here discuss our progress on understanding the different molecular mechanisms employed by cells and organisms to regulate autophagy in response to stressors such as aging and disease.

